# Variations of Saponins, Minerals and Total Phenolic Compounds Due to Processing and Cooking of Quinoa (*Chenopodium quinoa* Willd.) Seeds

**DOI:** 10.3390/foods9050660

**Published:** 2020-05-20

**Authors:** Manal Mhada, Mohamed Louay Metougui, Khadija El Hazzam, Kamal El Kacimi, Abdelaziz Yasri

**Affiliations:** 1 Laboratory of Natural Resources Valorization, AgroBioSciences, Mohammed VI Polytechnic University, Benguerir 43150, Morocco; Mohamed.METOUGUI@um6p.ma (M.L.M.); Khadija.ELHAZZAM@um6p.ma (K.E.H.); Aziz.YASRI@um6p.ma (A.Y.); 2 Laboratory of Bio-organic and Macromolecular chemistry, Department Chemical Sciences, Faculty of Science and Technology, Cadi Ayad University, Marrakech 40000, Morocco; 3 Industrial Executive Operations Division, Gantour Industrial Site, Act 4 Community Gantour, OCP, Youssoufia 46303, Morocco; kamal.elkacimi@ocpgroup.ma

**Keywords:** *Chenopodium quinoa*, processing, cooking, minerals, saponins, TPC

## Abstract

Quinoa (*Chenopodium quinoa* Willd.) is a grain of great nutritional interest that gained international importance during the last decade. Before its consumption, this grain goes through many processes that can alter its nutritional value. Here we report the effect of processing (polishing and milling) and cooking (boiling and steaming) on the saponin content, mineral profile of 14 elements using Inductively Coupled Plasma-Optical Emission Spectrometry (ICP-OES), protein content, and total phenolic compound. The polishing caused an average drop in the saponin content from 1.7% to 0.46% but induced important losses in mineral content (K, Mg, Ca, Zn, Co, Cu, Fe, Mn, and Ni), and phenolic compounds. However, the greatest nutritional degradation happened after milling due to the elimination of seed teguments and embryos, where over 50% of many minerals, 60% of protein content, and almost the totality of phenolic compounds, were lost. Cooking effect was less important than processing, but some significant losses were attested. Boiling caused a loss of up to 40% for some minerals like K, B, and Mo because of their hydrosolubility, and 88% of the polyphenols, while steaming allowed a better retention of those nutrients. Consuming polished quinoa instead of semolina and using steaming instead of boiling are trade-offs consumer needs to make to get optimal benefits from quinoa virtues.

## 1. Introduction

The Mediterranean diet has been subject to many studies bringing together health and environmental impact. It is mainly a plant-based diet with low consumption of animal products and thus has a smaller water footprint and lower greenhouse gas emissions, compared with other current diets [1,2]. Nevertheless, those assets are susceptible to change due to climate change. The Mediterranean region is becoming dryer and hotter with an annual increase of lands affected by salinity, making the cultivation of traditional crops harder and harder, which decreases the diversity in the field and thus in the plate [3], hence the interest of introducing new resilient and nutritive crops, like quinoa (*Chenopodium quinoa* Willd.) to new climate conditions.

Quinoa’s adaptability to extreme and diverse climatic conditions and its high nutritional value allowed the Food and Agriculture Organization (FAO) to support its introduction to several Mediterranean countries (Morocco, Algeria, Tunisia, Egypt, Lebanon, and Italy) through different initiatives focusing greatly on its adaptability to the local pedoclimatic conditions, diet, and cooking habits [4,5].

Quinoa originated from the Andes in South America. It has been part of the Andean diet for centuries and attracted the attention of scientists and industrials since 1975 [6]. Its grains are rich in fibers, minerals, vitamins, and a variety of phenolic, and antioxidant compounds. Furthermore, this pseudo-cereal contains on average 13% of proteins, sufficient amounts of all nine essential amino acids, and is naturally gluten-free with a low glycemic index [7]. As a result, many studies have shown that quinoa consumption may benefit several at-risk populations, including children, celiacs, people with diabetes, and cardiovascular disease-prone people, to list just a few [8,9,10].

However, quinoa grains are covered by a thin layer of glucoside compounds called saponins that gives a bitter taste to the grain and affects the product acceptance by the new potential consumers. This class of chemical compounds is widely present in over 100 families of both wild and cultivated plants [11]. Moreover, all saponins are potentially toxic if ingested in large quantities, because of their hemolytic activity, and therefore, there is a longstanding controversy about their functions in food [11]. Consequently, the grains are usually abraded and washed using different methods to remove their bitterness. Those different quinoa processing techniques lead to variations in grains composition regarding their properties and chemical constituents, producing grains with distinct nutritional characteristics [12].

In addition, to be fit for consumption, cooking the grains is mandatory. Processing and cooking alter the nutritional profile through many mechanisms (physical erosion, heat, and water solubility); thus, it becomes important to evaluate the effect of different processing and cooking methods on the nutritional value to maximize the benefit upon consumption.

The presented study aims to assess the effect of processing through polishing and milling (to produce quinoa’s semolina) and cooking via steaming and boiling on the saponin content, mineral profile, protein content, and total phenolic compound of two quinoa varieties.

## 2. Materials and Methods

### 2.1. Samples Origin, Preparation and Processing

**Figure 1:**
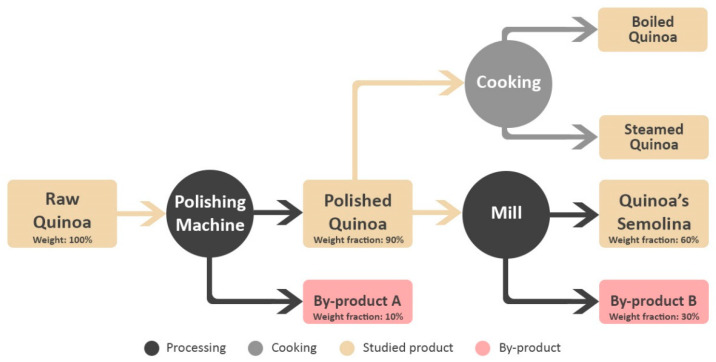
Studied quinoa processing and cooking diagram.

Quinoa samples were locally grown and obtained directly from a cereals and seeds producer located in Berrechid, Morocco (33° 12’ 48.03”N, −7° 51’ 7.22”W). Puno and Titicaca, the most cultivated quinoa varieties in Morocco, are the studied varieties. Samples were taken in triplicate after each step of the grain preparation process presented in Figure 1. To minimize the environmental effect on the seeds and unify the processing, all the samples were grown in the same year, taken from the same quinoa producer, and processed by one food company.

First, and before any treatment, the batch of raw grains was sampled. Then, and to be representative of the quinoa sold in the Moroccan market, the grains were polished using a custom mechanical grain polisher made by the quinoa producer to get rid of the saponin layer. Later, the grains were subjected to three separate treatments, described as follows.

The semolina was made by milling the grain trough a wheat mill, where its grooved steel rollers were adjusted so that the space between them is slightly narrower than the width of the quinoa grain resulting of quinoa’s coarse pieces (Semolina) separated through sifting in a 50 mm mesh sieve.

For steaming, the polished grains were transferred. For steaming, 100 g of polished quinoa was transferred to a stainless-steel steam pot, with 1 l of water, then placed on a heating plate for 45 min. Finally, for the boiled quinoa, the grains were put in a pan with water (1:3 v/v), kept boiling for 15 min, the time needed for a complete water evaporation, then any residual water was stained. After cooking, samples were collected, homogenized, and dried at room temperature.

All the dry samples were ground in a Waring 8010ES Blender (Waring Product Division, New Hartford, CT, USA) with a stainless-steel container and stored in sealed tubes at −20 °C until analysis.

### 2.2. Scanning Electron Microscopy Observations

The observed quinoa seeds were chosen to be representative of the processing level. For the cross-sections, cv. Titicaca seeds were prepared manually using a scalpel to visualize the different seed compartments and layers. Before observation, the samples were mounted on labeled metal stubs using a double-sided adhesive tape and then placed in an automatic carbon cord coater for 5 min. Magnification, resolution, and depth of focus were adjusted for each level of observation. No pretreatments were conducted on seeds since the samples were dry. The observations were made at different magnitudes using a Zeiss EVO 10 Scanning Electron Microscope (SEM) at a voltage of 20 Kv (Oberkochen, Germany).

### 2.3. Chemical and Nutritional Analysis

#### 2.3.1. Mineral Profile

After grinding, 500 ± 0.5 mg of quinoa samples were weighted in triplicate and placed in a digestion tube for the mineralization as a first step, then 7.5 mL of HNO_3_ 65% was added. The tubes remained open for 20 min to avoid a process interruption due to a rapid increase in pressure. Then, they were closed and positioned inside the microwave rotor and digested for 2 h at 90 °C. The final volume was adjusted to 50 mL with deionized water. For the second step, the final solution was quantitatively transferred to a polypropylene tube and filled up to 10 mL.

A multi-elemental trace analysis of previously digested quinoa samples was carried out using the Agilent 5110 ICP-OES (Santa Clara, California, USA). The analyzed elements were K, P, S, Mg, Ca, Na, Fe, Zn, Mn, B, Cu, Ni, Co, and Mo. TraceCERT^®^ mono-element ICP standards with a concentration of 1000 mg/L each in nitric acid obtained from Merck (Darmstadt, Germany), were used. Calibration standards were prepared from stock solutions and stored at 4 °C.

#### 2.3.2. Proteins Content Determination

Protein content was measured using the Kjeldahl method for organic nitrogen. The total nitrogen content of the studied samples was determined according to the official methods of analysis of AOAC International (Association of Official Analytical Chemists) using a Kjeltec 2300 autoanalyzer (Hilleroed, Denmark) [13]. Samples of 0.3 g of powdered quinoa were placed in a digestion tube with 10 mL of added sulfuric acid and salicylic acid. The tubes were then placed in a Kjeldahl catalyzer and heated to 380 °C for 2 h until the samples turned hyaline. Total Kjeldahl nitrogen, which is the sum of organic nitrogen (N), ammonia (NH3), and ammonium (NH4^+^) was determined by distillation. The measurements of each treatment were made in triplicates. The obtained nitrogen content was converted into a protein content using quinoa’s specific conversion factor determined by Fujihara et al., (2008) of 5.75 [14].

#### 2.3.3. Saponin Content and Total Phenolic Compounds (TPC) Determination

The extractions of both saponins and total phenolic compounds were based on Navarro del Hierro et al., (2018) with modifications [15]. In short, 5 g of each sample’s powder was defatted using Soxhlet method with petroleum ether as solvent. An ultrasound-assisted extraction using pure methanol (1:10 *w*/*v)* was carried out with an ultrasonic probe at a 60% amplitude for 15 min. The mixture was then centrifuged at 3400 rpm for 10 min. The supernatant was collected and completed to 50 mL with methanol.

The saponin content determination was carried out according to the method applied by Irigoyen et al., (2018), by adding 1 mL of the diluted extract (1:5 dilution) to 3.5 mL of the Lieberman-Buchard reagent (16.7% acetic anhydride in concentrated sulfuric acid) [16]. The solution was vortexed and stored in the dark for 30 min at room temperature. The absorbance of the solution was measured at 528 nm in a spectrophotometer. Oleanolic acid was used as a standard to prepare a calibration curve. The results were expressed in g of saponins per 100 g of dry matter.

For the TPC content, it was estimated using the Folin-Ciocalteu’s (F-C) reagent method [17]. Briefly, 200 µL of each sample was mixed with 1 mL of F-C reagent (freshly diluted in a 1:10 proportion with distilled water), allowed to react for 5 min at room temperature in the dark, and then 800 µL of 7.5% anhydrous sodium carbonate solution (*w*/*v*) was added. The mixture was incubated for 30 min in the dark prior before measuring the absorbance at 750 nm using a spectrophotometer. Gallic acid (GA) was employed as a reference standard to prepare the calibration curve. The results were expressed as mg GA 100 g of dry matter.

### 2.4. Statistical Analysis

The gathered data of the 14 elements, proteins, saponins, and TPC was separated into two matrices. A first matrix for quinoa processing, grouping the data of raw quinoa, polished quinoa, and quinoa’s semolina, while the second for quinoa cooking, gathering the data of polished, boiled, and steamed quinoa. For both tables, means and standard deviations were calculated by treatment and by variety. To compare between quinoa treatments, varieties, and their interaction, a two-way analysis of variance was performed using the general linear model. When the residuals were not normally distributed or did not have a homogeneous variance, the ANOVA model was adjusted using the Box-Cox method with an optimal λ. Then, Tukey’s multiple comparison test (*p* < 0.05) was applied to discriminate statistically different values.

Descriptive statistics, data transformation, analysis of variance and comparison of means were carried out using the software R version 3.6.0 (Foundation for Statistical Computing, Vienna, Austria), with the *Agricolae* package version 1.3-2 and R’s MASS package version 7.3–51.3 [18,19]. The graphs were generated using Microsoft Excel version 16.0 and R version 3.6.0.

## 3. Results and Discussion

### 3.1. Effect of Processing on Quinoa Grains

In the first part of the present study, the effects of polishing and milling on macro and micro-elements, proteins, saponins, and total phenolic compounds (TPCs) are investigated. Table 1 presents, the average concentration of 14 minerals determined using ICP-OES, proteins, TPC and saponins for both studied quinoa varieties and each grain state.

#### 3.1.1. Effect of Processing on Saponin Content

**Figure 2:**
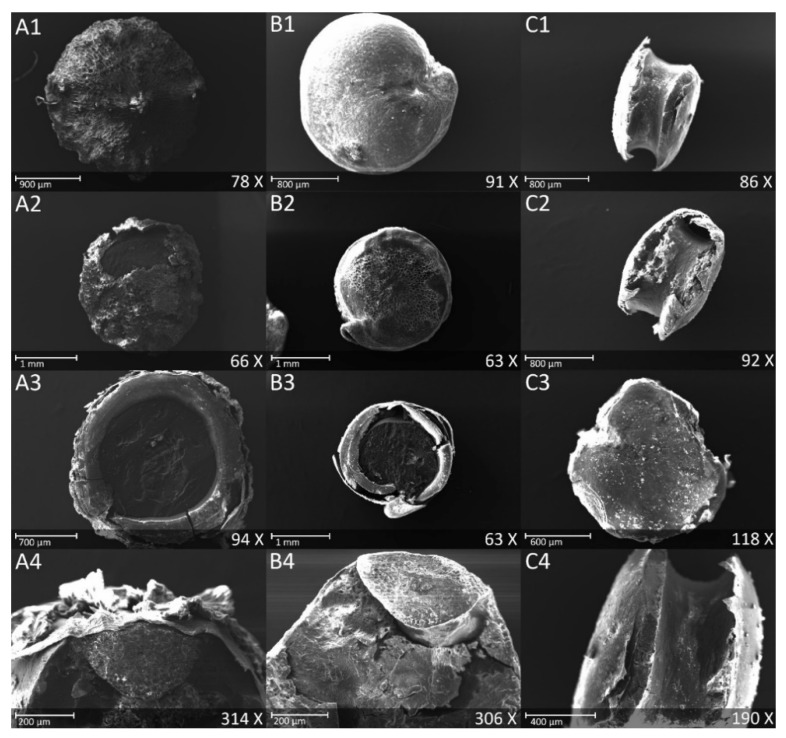
Scanning Electron Microscope images of quinoa seeds cv Titicaca. (**A**) Raw seeds: (**1**) Dorsal surface, (**2**) Ventral surface, (**3**) Cross section showing different seed layers, (**4**) Longitudinal section showing the embryo. (**B**) Polished seeds: (**1**) Dorsal surface, (**2**) Ventral surface with residual outer layer, (**3**) Cross section showing the embryo and the seed layers, (**4**) Longitudinal section showing the embryo. (**C**) Semolina: (**1**) Lateral view, (**2**) Lateral view showing residual layers and (**3**) Dorsal surface, (**4**) Lateral view showing the empty location of the embryo.

Lowering the initial saponin content and improving the taste is the main reason for quinoa polishing and processing. The polishing detaches the remaining perianth (Figure 2(A1,A2)), a panicle residue that plays a protective role to the seeds during maturation and storage [20].

In addition, it is applied to abrade the indehiscent pericarp, a dry protective layer made of papillose cells derived from the outer epidermis of the ovary cells, and containing most of the grains’ saponins [21]. In our case, the industrial abrasion achieved the described role as can be seen comparing Figure 2A,B, where they show that most of the outer layers have been abraded. This mechanical polishing allowed the reduction of saponins level from 2% to 0.42% for Titicaca, and from 1.4% to 0.51% for Puno, a reduction of 80%, and 64% of the initial saponin level respectively (Table 1). These results are aligned with other studies showing that mechanical polishing can decrease saponins level between 50% and 85%, but this depends on many factors, including milling degree and seeds initial saponin content [22,23,24].

The varieties we are investigating are classified as bitter [25], and the important reduction observed in saponins level by polishing was not enough to classify the quinoa as sweet, since the threshold of human saponins detection is 0.11% [26], hence the need to wash the grains to get rid of the residual saponins, since a more extensive polishing will increase seeds defection through breaking and damaging [22].

Regarding the saponins in quinoa’s semolina, a highly processed product to our knowledge never studied before, they were almost nonexistent (Table 1). In fact, the important level of milling and sieving produced small semolina grains consisting mainly of the perisperm alone, as observed in Figure 2(C1). In Figure 2(C3), representing a cross-section of the semolina grain, we can see that besides the lack of the embryo, an important part of the external layers was abraded compared to the polished grain, especially the seed coat the remaining layer in the polished quinoa observed in Figure 2(B3). Therefore, the level of saponins found is expected since Ando et al., (2002) found that quinoa perisperm contain only 3% of the total whole seeds saponin content [24].

#### 3.1.2. Effect of Processing on Mineral Profile and Protein Content

The mineral fraction constituted 2.0%, 1.4%, and 0.8% of the raw grain, polished grain, and semolina’s total weight respectively, showing a clear degradation of the food mineral richness with the processing (Table 1). Between varieties, the whole grain mineral profile and protein content were closely similar except for Fe and Zn where the values were significantly (*p* ≤ 0.05) higher for the variety Puno (Table 1). This ionomic profile similarity is explained by the fact that both varieties were developed for the Mediterranean climate in Denmark in 1988 and cultivated in the same environment [27].

Comparing elements by proportion, K was the major macro-element, constituting on average 50% of the total mineral fraction of the three quinoa forms and in both varieties. The remaining macro-elements, by abundance, were P, followed by S, Mg, and Ca, then Na (Table 1). For micro-elements, Fe was by far the most abundant mineral with a concentration in raw seeds of 83 mg/kg for Puno and 58 mg/kg for Titicaca, followed in descending order by Zn, Mn, B, Cu, Ni, Co, and Mo. Those findings are nearly the same as found in literature where K and P are the dominant macro-elements followed by Mg, then Ca, and by abundance Fe, Zn, Mn, and Cu are the main microelements, and Ni, and Mo occur in trace [24,28,29]. However, the absolute concentrations can change quite much between our study and many other studies since the seeds were not grown in the same environment, nor used the same genetic material. The quinoa grown in Morocco has a higher K, Ca, and Mg than that reported in most of the other studies interested in quinoa’s mineral profile [24,28,29]. These values are probably the result of the calcareous soil rich K, Ca, and other elements, but this assumption needs to be confirmed by further agronomical studies.

**Figure 3:**
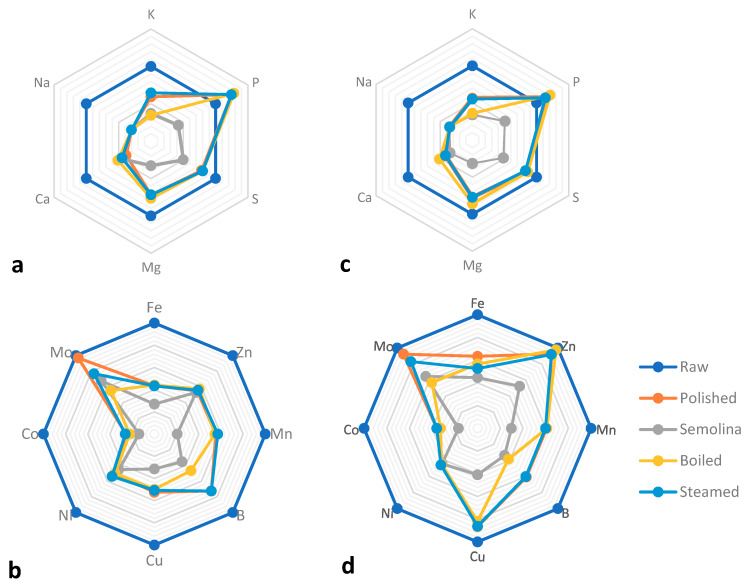
Macro and micro-minerals as % of their concentrations in raw seeds. With (**a**,**b**) for Puno and (**c**,**d**) for cv Titicaca.

Comparing unprocessed, polished, and milled grains, the results showed that all the minerals were significantly different between the three forms of the grain, except for Mo, Ni and Na. In fact, Mo remained stable between all three quinoa forms, while Ni and Na levels remained the same between polished grains and semolina (Table 1). For macro-elements, and for both varieties, the polished grains maintained approximately 75%, 60%, 40%, and 30% of whole grains Mg, K, Ca, and Na respectively. While the content of P increased by 20% and S decreased by the same level (Table 1, Figure 3).

Regarding micro-elements, the greater change was observed in Co, where the polished grains preserved on average only 30% of the quantity observed in raw quinoa. Followed by Ni, Fe, Mn, and B where only 48%, 53%, 58%, and 67% of the original content were maintained, respectively.

For Zinc’s and Copper’s preservation, it depended on the variety. In fact, Zn change in Titicaca was minimal with 29.37 mg/kg for raw seeds and 27.17 mg/kg for polished grains (Figure 3d), while for Puno, in processed grain Zn represented only 55% of non-treated grain concentration of this mineral (Table 1, Figure 3b). For Cu, Tukey’s test showed that unpolished and polished grains had the same content in Titicaca, while it dropped from 5.90 mg/kg to 3.10 mg/kg in Puno (Table 1, Figure 3b).

Our results are partially in concordance with the literature. Ando et al., (2002) found that polished quinoa had lower K, Mg, Ca and higher P [24]. However, the authors also found that Fe, Zn, Cu, and Mn content stayed stable [24]. These differences, especially for Fe and Mn, can be due to the differences in the used genetic material since they worked with var. Real, or can result from a lower degree of polishing since the machines used for quinoa processing are not the same [24].

For quinoa’s semolina, the degradation was very important, and the mineral content was less than 50% compared to that of the unprocessed grains mainly due to the high abrasion level that reached the embryo (Figure 2(C1–C4)). The major losses were observed in Co and Mn where their content in semolina was less than 30% of that of the whole quinoa. Mg, B, K, Fe, and Cu content was less than 40% and for the remaining the content was between 40% and 50%. The only element that kept 66% of the whole grain initial concentration was Zn (Table 1, Figure 3).

Regarding the protein content, an important macronutrient in quinoa, it wasn’t affected by the polishing or the varietal aspect. In fact, the raw and polished quinoa were not statistically different with an average of 12.75 g/100 g (Table 1). The protein content results are in line with many other studies stating that quinoa’s protein content is between 11 and 16 g/100 g [30]. For semolina, the protein content was on average 5.75 g/100 g. This low protein content is mainly the result of the milling process detaching the embryo from the seed which is a protein-rich component (Figure 2(C1–C4)), and keeping essentially the perisperm, a seed compartment attested in literature to have a protein content of 7.2 g/100 g using a conversion factor of 6.25 [24].

#### 3.1.3. Effect on Total Phenolic Compounds

The phenolic compounds are secondary metabolites that are widely shown to have many potential health benefits [31,32]. Table 1 shows that for quinoa the phenolic compound is widely affected by genotype and processing. The TPC of Puno was 31.7 mg GA/100 g while for Titicaca it was 106 mg GA/100 g. The superior TPC in Titicaca compared to Puno could be explained by the seed color which is darker for Titicaca. This assumption can be corroborated with the results found by Han et al., (2019) on a study about the TPC on seven quinoa varieties where they found that darker quinoa varieties had a higher content of phenolic compounds, as well as higher flavonoids and antioxidant activity [33]. Compared to literature, the TPC values of both varieties were lower than those found by Han et al., (2019) where the authors found that non-processed quinoa TPC content was 200.4 mg GA/100 g in a Chinese cultivar [23]. However, they were at the same order as other studies, where Nickel et al., (2016) found that TPC content of Brazilian quinoa variety was 97.6 mg GA/100 g, Alvarez-Jubete et al., (2010) found a TPC content of 71.7 mg GA/100 g in Bolivian quinoa, and Miranda et al., (2010) a content of 28.4 mg GA/100 g in Chilean cultivars [34,35,36]. This shows the complexity of the synthesis of these compounds by the plant affected by the genotype and environment.

Regarding the TPC content in processed quinoa, no statistical differences were found between polished and raw seeds for var. Puno, while for var. Titicaca, the concentration in polished quinoa seeds was nearly 60% (67.9 mg GA/100 g) of that of the whole seeds (Table 1). For quinoa’s semolina, the difference of TPC content between the two varieties was non-significant, with an average of 0.16 mg GA/100 g (Table 1). The phenolic compound degradation with processing was attested in other studies, where Gómez-Caravaca et al., (2014) found a 21.5% decrease in TPC trough quinoa pearling, and Han et al. (2019) found that with a degree of milling of 27.23%, the grains maintained only 68% of the TPC content [15]. In addition, this very high TPC degradation after milling suggests that this family of secondary metabolites is mainly present in the outer layers of the seed and the embryo while absent from the perisperm.

#### 3.1.4. Quinoa Nutrients Fractioning Through Processing

To estimate the absolute amount of lost nutrients per processing phase and with each byproduct generated, weight fractioning was obtained from the processing company. A quantity of 100 g of whole quinoa produces on average 90 g of polished grains and 10 g of by-product A, a by-product containing most of the whole grain’s saponins. Then, the 90 g of the produced polished grains gives 60 g of semolina and 30 g of by-product B (Figure 1).

**Figure 4:**
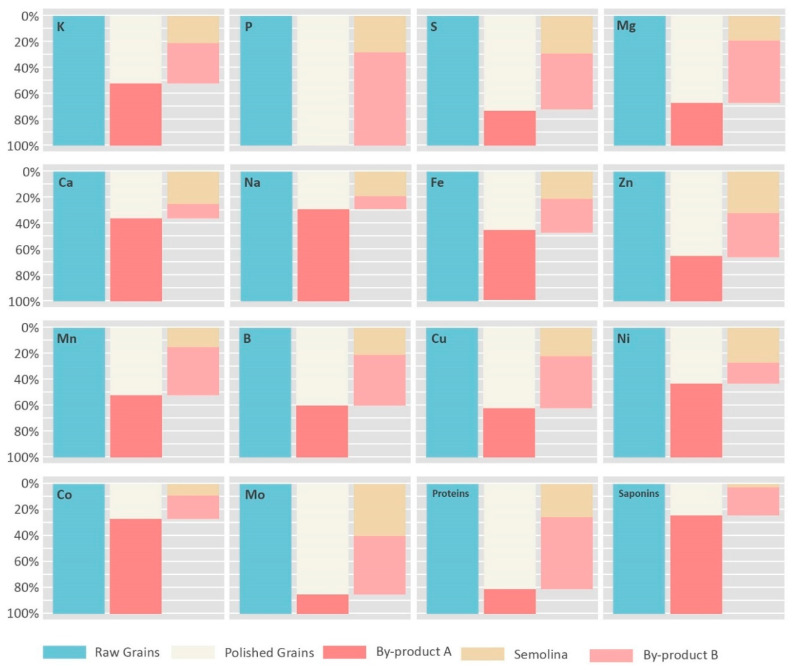
Minerals, proteins, and saponins proportions in different quinoa fractions and by-products generated through the different processing forms.

Figure 4 presents the relative proportion of minerals and protein content in each fraction. For minerals in the polishing phase, apart from P, and Mo all the elements fractions lost with byproduct A were superior to its weight fraction (10% of the raw seeds total weight), explaining the loss in concentration of most elements in the polished grains (Table 1). By abundance, Co, Na, Ca, Ni and Fe were the most affected elements with over 50% of their initial quantity lost with byproduct A. In addition, Mn, K, B, Cu, Zn, and Mg presented high erosion levels ranging between 48% and 33%. This suggests a high concentration of these elements in the outer layers of the grain. These results agree with the element mapping through EDX of Mg, P, K, Ca, and S done by Konishi et al., (2004), where he found that Ca mostly exists in the pericarp, and that K, and Mg exists with a high concentration in the pericarp and embryo. In addition, he showed that P exists with a large concentration in the embryo and is absent in the pericarp, explaining the absence of P in by-product A [37].

The production of quinoa’s semolina, a sweet and more adapted product to Mediterranean cuisine, needs an additional milling phase. As mentioned above, from the remaining weight of polished grain 2/3 remains in the semolina and 1/3 is lost to by-product B. Other than Ca, Ni, and Na, which represented nearly the 2/3 of polished grain quantity of these elements, explaining the stability of the concentration of these elements between polished grain and semolina (Table 1), all the remaining elements were subjected to important losses with by-product B. In fact, most losses were observed in P, Mn, and Mg with more than 70% of the quantity that remained in the polished grain after the first processing (Figure 4). In addition, by abundance, between 66% and 50% of Co, B, Cu, S, K, Fe, and Zn polished quinoa quantity was lost with by-product B (Figure 4). These important losses are explained by the loss of embryos after removing nearly 40% of the raw grain mass, which can be observed in Figure 2(C1–C4).

The embryo is a seed compartment rich in many nutrients, and its loss causes the observed reduction in many elements. In fact, its degradation was also the main cause of the protein loss observed in the semolina, where 75% of the raw grain’s protein quantity and nearly the totality of TPC was lost to both byproducts (Figure 4). Many studies focusing on the location of nutrients and reserves in quinoa seeds found that the proteins exist with a high percentage in the embryo, and according to Ando et al., (2002), it constitutes around 57% of the total grain proteins [24].

From the fractioning illustrated Figure 4, and the comparison between unprocessed and processed forms of quinoa, we can conclude that an important part of the nutrients is lost in the by-products, therefore their valorization is a necessity. Table 2 presents estimations of the content of minerals, proteins, TPC, and saponins in both by-products calculated from the losses observed between the 3 studied quinoa forms.

Both byproducts are rich in nutrients compared to the raw grains. For minerals, byproduct A is very concentrated in all elements, but for P, which is almost absent; for the rest of macro and microelements they are for the most part 3 to 6 folds more concentrated than in raw grains. In addition, the concentrations of some elements can change considerably depending on variety, like Fe, Zn, or Cu where respectively their content is 505.2, 163.9, and 31.1 mg/kg in Puno and 247.7, 49.2, and 12.0 mg/kg in Titicaca (Table 2). For byproduct B, it is less concentrated in minerals than byproduct A, but is richer than raw quinoa for most elements especially P which is 2.5 times the whole grain’s concentration for both varieties. Regarding proteins, the content is between 21.7 to 29.3 g/100 g in both by-products showing a concentration of 1.5 to 2 times greater than the grain (Table 2). While for TPC, it depends on the variety, where for Puno, the less concentrated variety in these metabolites, both by-products had a content of around 79 mg GA/100 g compared to 31.67 mg GA/100 g raw grains, while for Titicaca, the concentration is 447.8, and 203.0 mg GA/100 g for by-product A, and B respectively, compared to 105.85 mg GA/100 g for raw grains of the same variety (Table 2).

These results show the richness of quinoa’s production line byproducts, but their valorization will depend mainly on the saponin content. Even being the more nutritive of both byproducts, byproduct A contains a high concentration of saponins (9.5% for Puno, and 16.5% for Titicaca), which limits the possibilities of its use in the food industry and can be more adapted to be used in cosmetic, pharmaceutical, or agronomical industries (Table 2). However, byproducts B, and even with a relatively high saponin level (1.4% for Puno, and 1.1% for Titicaca), can be used to fortify other products, like gluten free flour or other mixes, which will dilute the saponin content while taking benefit of the byproduct’s richness (Table 2).

### 3.2. Effect of Cooking on Quinoa Grains

Cooking is as important as quinoa processing since it is a needed step before this grain consumption. Like processing, results show that this crucial step influences the nutrient content of this aliment. Table 3 presents the average concentration of 14 minerals, proteins, TPC, and saponins for both studied cooked forms of quinoa compared to uncooked grains and for both varieties.

#### 3.2.1. Effect of Cooking on Saponin Content

Even if largely removed during quinoa processing, saponins levels are still superior to the human detection threshold (>0.11%) as discussed in the previous chapter. The cooking process helped reduce these levels but not to the same extent since the saponin level was on average 0.2% in steamed quinoa, where it was only 0.06% in boiled one (Table 3). This important reduction during boiling is due to the leaching of saponins from the seeds to the cooking water and their elimination after straining the remaining water. In fact, quinoa saponins contain a large group of glycosides that are soluble in water, and causes solutions foaming [38]. For steaming, the seeds do not come in direct contact with water, so the soluble saponins leach slowly to ambient humidity inside the steaming pot, but the transfer from the grains to water stays limited.

These results show that the water can remove the remaining saponins from the polished grains. In addition, that a thorough washing is necessary especially before steaming, since cooking without washing the grains keeps the saponin level above the human detection threshold.

#### 3.2.2. Effect of Cooking on Mineral Profile and Protein Content

The usual way to prepare quinoa is to boil it in water. This cooking technic has the advantage of being quick, softening the grain, and disposing of the residual saponins that remain on the seeds. Unfortunately, results showed that this way of cooking induces a significant loss in some minerals, compared to steaming, especially those hydrosoluble (Table 3).

In quinoa, the embryo, a seed component naturally rich in nutrients in all species, is located around the seed. In addition, the protective layer that covers it, is removed after polishing, which facilitates the leaching of hydrosoluble minerals from the seed to the outer aqueous medium.

In our case, boiling caused significant losses (*p* ≤ 0.05) in K, that was reduced by 41%, B with 36%, and Mo with 43% (Table 3). This is directly related to the solubility of these elements, especially K that was reported in many studies to decrease in food after boiling [39]. However, steaming seems to be more element conservative. In fact, all the minerals remained the same after steaming, except for Ca, where in Puno a significant increase has been observed in cooked quinoa compared to uncooked, but this difference was non-significant (Table 3).

Based on these results, and for both studied varieties, the mineral profile stays quite stable for both cooking methods, but for some elements. However, the steaming has clearly a higher retention index for the hydrosoluble minerals compared to boiling.

Concerning proteins, no significant differences were observed between the uncooked and cooked grains, hence the protein richness of the food stays stable after cooking (Table 3).

#### 3.2.3. Effect of Cooking on Total Phenolic Compound

The results show that cooking caused an important degradation of the TPC (Table 3). The amounts of phenolic compounds in steamed quinoa samples were higher than the boiled samples with a retention level respectively for Puno and Titicaca of 8% and 13% in boiled samples, compared to 41% and 24% for the steamed samples. Losses of the TPC can be explained by thermal degradation and the release of these compounds into cooking water. In fact, in a quinoa cooking experiment trough boiling, Dini et al. (2010) found that an important part of quinoa TPC is lost to boiling water, but a part broke down and was non-detectable through spectrophotometry, since from the initial concentration of 77.2 mg GA/100 g in uncooked seeds, only 28,7 mg GA/100 g remained after cooking and 14 mg GA/100 g were retrieved in cooking water [40]. However, in another study, Nickel et al., (2016) found that the TPC increase after cooking and explains this by the release of the soluble phenolic compound which makes them more detectable. It is also noteworthy that in this study, no water remained after cooking the quinoa, which prevented the TPC leaching [34].

In our study, even though degradation and leaching explain most of the TPC losses, the retention level of these molecules was very low compared to the other studies. This is probably the result of the additional drying performed in the sample preparation which caused an additional degradation of the polyphenols and their binding to other compounds. As found by Miranda et al., (2010) in a quinoa air drying experiment, where they showed that an important TPC degradation happens with the seeds drying [36].

### 3.3. Contribution of Quinoa to the Recommended Daily Intake

Quinoa seeds are one of the best sources of energy, complex carbohydrates, plant proteins, fats, vitamins, minerals, and other important biologically active compounds. The evident benefits for health necessitate the inclusion of quinoa products in the nutrition of both adults and children. In addition, the intake of quinoa-based products is associated with a lower risk of cardiovascular diseases, diabetes, obesity, colorectal cancer in adult patients [8].

Quinoa’s nutritional value should never be directly computed from the whole grain values, since as demonstrated in this study many nutrients are lost from the whole grain to the consumed form. Consequently, the contribution of quinoa to the recommended daily intake (RDI), which is the amount of proteins, vitamins, and minerals that should be consumed daily to meet the nutritional needs of most individuals in a healthy population, should be calculated from the ready-to-use forms. Table 4 represents the relative contribution of 50 g of boiled quinoa, steamed quinoa, quinoa’s semolina to the Recommended Dietary Allowance (RDA), Adequate Intake (AI), and the Tolerable Upper Intake Level (UL) levels prescribed by WHO (World Health Organization) and FAO (Food and Agriculture Organization), for the studied nutrients.

The contribution of cooked quinoa to the RDI is very interesting for all categories, especially P, Mg, Mn, and Cu. In fact, we can see that a proportion of 50 g of cooked quinoa can provide around 63% of Mn, 62% of Mg, 33% of Cu, and 32% of P to a child RDI of those elements. In addition, the data shows that the quinoa is a good source of Fe and Zn, where 50 g provides more than 18% of a child’s RDI, and more than 10% of a woman’s RDI for these elements (Table 4). Furthermore, if the objective is to get more K, it will be preferable to consume steamed quinoa rather than boiled. For the proteins, quinoa remains a good source, because while 50 g responds to around 10% of an adult male RDI, it gives at the same time all the essential amino acids, as opposed to other cereals and legumes (Table 4).

Finally, when compared to wheat, an important cereal in the Mediterranean diet, polished quinoa provides nearly the same intake for minerals, except for K and Ca, where the same proportion provides two times the RDI for these two minerals [41]. When compared to other gluten free cereals like white and brown rice, quinoa provides a superior amount of nearly all the elements and proteins. Compared to brown rice, one of the most nutritive types of rice, quinoa provides two times the K, 1.5 times the Mg, 10 times the Ca, 2.5 times the Fe, and more than two times the Cu [42]. In addition, the semolina, even if less nutritious than the whole quinoa, remains richer than white rice for most minerals [42].

The Mediterranean diet has been associated historically with good health. It is characterized by the abundance of plant-based foods as a core of the daily intake. For instance, in North Africa, couscous is one of the most balanced dishes in the world’s traditional recipes, where it is based on a small quantity of meat, diverse seasonal legumes, and semolina. The introduction of new nutritive grains like quinoa to those cuisines can enrich them even more.

## 4. Conclusions

This study showed the existence of important losses in some elements, especially calcium, and that without reducing the saponin level below the upper limit of human saponin detection. Thus, developing alternative methods, probably combining dry and wet saponin reduction technics, could be the answer to reduce the saponin level to required limits without losing much in terms of nutritional value. Regarding quinoa’s semolina, it presents the lowest level of saponins, but unfortunately it loses an important part of its nutritional value. Nevertheless, its contribution to the RDI stays more important than some widely used cereals like white rice. Regarding the cooking itself, it does not alter very much the mineral profile except for some elements like potassium and boron. Steaming happens to be more conservative than boiling, thus it is recommended to use it more for quinoa preparation.

Like many studies focusing on the genetic diversity of quinoa, we found that genotype plays an important role in the grain nutritional value, and that even when they are both grown in the same environment. Consequently, choosing the right variety and growing it in the right environment and with the optimal soil nutrition program is as important as the post-harvest processing. Thus, more studies exploring the impact of those conditions on each fraction of the grain nutrients should be undertaken.

## Figures and Tables

**Table 1:** Means and standard deviation of saponin content, mineral concentrations, protein content, and Total Phenolic Compounds in raw and processed quinoa seeds of cv. Puno and cv. Titicaca.

Nutrient		Raw		Polished		Semolina	
		Puno	Titicaca	Puno	Titicaca	Puno	Titicaca
Saponins (%)		1.41 ± 0.05^b^	2.03 ± 0.29^a^	0.51 ± 0.09^c^	0.42 ± 0.04^c^	0.09 ± 0.01^d^	0.07 ± 0.01^e^
Macro-elements (g/kg) (LOQ)	K (0.003)	11.87 ± 1.74^a^	10.27 ± 0.78^a^	7.03 ± 0.23^b^	5.80 ± 0.36^b^	4.40 ± 0.00^c^	3.50 ± 0.26^d^
	P (0.003)	2.60 ± 0.17^b^	2.70 ± 0.00^b^	3.23 ± 0.23^a^	3.10 ± 0.00^a^	1.10 ± 0.00^d^	1.37 ± 0.06^c^
	S (0.001)	2.00 ± 0.17^a^	1.93 ± 0.12^a^	1.57 ± 0.12^b^	1.63 ± 0.12^ab^	1.00 ± 0.00^c^	0.93 ± 0.06^c^
	Mg (0.001)	2.13 ± 0.21^a^	1.90 ± 0.10^a^	1.53 ± 0.06^b^	1.47 ± 0.06^b^	0.70 ± 0.00^c^	0.60 ± 0.00^d^
	Ca (0.01)	1.57 ± 0.25^a^	1.43 ± 0.06^a^	0.60 ± 0.00^c^	0.60 ± 0.00^c^	0.73 ± 0.06^b^	0.50 ± 0.00^d^
	Na (0.003)	0.67 ± 0.15^a^	0.57 ± 0.06^a^	0.20 ± 0.00^b^	0.20 ± 0.00^b^	0.20 ± 0.00^b^	0.20 ± 0.00^b^
Micro-elements (mg/kg) (LOQ)	Fe (0.5)	83.05 ± 5.16^a^	57.62 ± 6.15^b^	36.14 ± 1.85^c^	36.5 ± 2.00^c^	22.36 ± 0.40^d^	25.71 ± 2.71^d^
	Zn (0.5)	32.33 ± 0.98^a^	29.37 ± 0.29^b^	17.72 ± 0.54^d^	27.17 ± 0.32^c^	17.12 ± 0.25^d^	15.39 ± 0.45^e^
	Mn (0.5)	34.43 ± 4.93^a^	29.71 ± 1.72^a^	19.07 ± 0.79^b^	18.07 ± 1.22^b^	7.13 ± 0.03^c^	8.85 ± 0.85^d^
	B (0.5)	10.14 ± 0.36^a^	8.58 ± 0.74^ab^	7.36 ± 0.48^b^	5.22 ± 0.38^c^	3.58 ± 0.14^d^	2.89 ± 0.09^e^
	Cu (0.5)	5.90 ± 0.51^a^	5.41 ± 0.05^ab^	3.10 ± 0.17^c^	4.68 ± 0.20^b^	1.86 ± 0.05^e^	2.21 ± 0.07^d^
	Ni (0.05)	1.40 ± 0.32^a^	1.11 ± 0.05^a^	0.71 ± 0.05^b^	0.50 ± 0.03^c^	0.64 ± 0.02^b^	0.49 ± 0.01^c^
	Co (0.05)	0.43 ± 0.06^a^	0.30 ± 0.01^a^	0.11 ± 0.02^b^	0.11 ± 0.01^b^	0.06 ± 0.00^c^	0.05 ± 0.00^d^
	Mo (0.05)	0.22 ± 0.02^a^	0.22 ± 0.07^a^	0.21 ± 0.00^a^	0.20 ± 0.06^a^	0.15 ± 0.01^a^	0.14 ± 0.05^a^
Proteins (g/100 g)		13.41 ± 0.72^a^	13.43 ± 0.81^a^	11.64 ± 0.65^a^	12.52 ± 0.67^a^	5.9 ± 0.21^b^	5.58 ± 0.35^b^
TPC (mg GA/100 g)		31.67 ± 7.26^c^	105.85 ± 5.21^a^	26.31 ± 0.78	67.86 ± 1.62^b^	0.05 ± 0.28^d^	0.28 ± 0.74^d^

Mean values in the same line sharing the same letter do not differ significantly at *p* < 0.05. TPC: Total Phenolic Compounds, LOQ: limits of quantification.

**Table 2:** Estimated content of saponins, minerals, proteins and Total Phenolic Compounds (TPC) in quinoa production line byproducts (Bp) by variety.

	Byproducts	Byproduct-A		Byproduct-B	
	Nutrients	Puno	Titicaca	Puno	Titicaca
	Saponins (%)	9.5	16.5	1.4	1.1
Macro-elements (g/kg)	K	55.4	50.5	12.3	10.4
	P	0.0	0.0	7.5	6.6
	S	5.9	4.6	2.7	3.0
	Mg	7.5	5.8	3.2	3.2
	Ca	10.3	8.9	0.3	0.8
Micro-elements (mg/kg)	Fe	505.2	247.7	63.7	58.1
	Zn	163.9	49.2	18.9	50.7
	Mn	172.7	134.5	42.9	36.5
	B	35.2	38.9	14.9	9.9
	Cu	31.1	12.0	5.6	9.6
	Ni	7.6	6.6	0.9	0.5
	Co	3.3	2.0	0.2	0.2
	Mo	0.3	0.4	0.3	0.3
	Protein (g/100 g)	29.3	21.7	23.1	26.4
	TPC (mg GA/100 g)	79.8	447.8	78.8	203.0

TPC: Total Phenolic Compounds, LOQ: limits of quantification.

**Table 3:** Means and standard deviations of mineral concentrations, proteins content, TPC, and saponins content in boiled and steamed quinoa seeds.

Nutrient		Polished		Boiled		Steamed	
		Puno	Titicaca	Puno	Titicaca	Puno	Titicaca
Saponins (%)		0.51 ± 0.09a	0.42 ± 0.04a	0.06 ± 0.01c	0.06 ± 0.00c	0.19 ± 0.01b	0.22 ± 0.03b
Macro-elements (g/kg) (LOQ)	K (0.003)	7.03 ± 0.23a	5.80 ± 0.36b	4.10 ± 0.00c	3.70 ± 0.20c	7.67 ± 0.15a	5.67 ± 0.35b
	P (0.003)	3.23 ± 0.23a	3.10 ± 0.00a	3.33 ± 0.06a	3.27 ± 0.06a	3.23 ± 0.15a	3.07 ± 0.06a
	S (0.001)	1.57 ± 0.12a	1.63 ± 0.12a	1.60 ± 0.00a	1.67 ± 0.15a	1.60 ± 0.00a	1.60 ± 0.17a
	Mg (0.001)	1.53 ± 0.06ab	1.47 ± 0.06b	1.63 ± 0.06a	1.63 ± 0.06a	1.53 ± 0.06ab	1.47 ± 0.06b
	Ca (0.01)	0.60 ± 0.00c	0.60 ± 0.00c	0.80 ± 0.00a	0.73 ± 0.06b	0.70 ± 0.00b	0.60 ± 0.00c
	Na (0.003)	0.20 ± 0.00a	0.20 ± 0.00a	0.20 ± 0.00a	0.20 ± 0.00a	0.20 ± 0.00a	0.20 ± 0.00a
Micro-elements (mg/kg) (LOQ)	Fe (0.5)	36.14 ± 1.85a	36.50 ± 2.00a	36.66 ± 0.38a	32.65 ± 2.75a	36.00 ± 1.55a	30.33 ± 3.52a
	Zn (0.5)	17.72 ± 0.54b	27.17 ± 0.32a	18.59 ± 0.08b	28.66 ± 1.27a	18.03 ± 0.40b	26.98 ± 0.20a
	Mn (0.5)	19.07 ± 0.79a	18.07 ± 1.22a	18.81 ± 0.22a	18.16 ± 1.47a	19.69 ± 0.91a	17.79 ± 0.98a
	B (0.5)	7.36 ± 0.48a	5.22 ± 0.38b	4.73 ± 0.09b	3.30 ± 0.25c	7.37 ± 0.23a	5.16 ± 0.35b
	Cu (0.5)	3.10 ± 0.17b	4.68 ± 0.20a	2.94 ± 0.05b	4.44 ± 0.25a	2.99 ± 0.07b	4.67 ± 0.15a
	Ni (0.05)	0.71 ± 0.05a	0.50 ± 0.03b	0.70 ± 0.08a	0.50 ± 0.05b	0.76 ± 0.05a	0.51 ± 0.04b
	Co (0.05)	0.11 ± 0.02a	0.11 ± 0.01a	0.10 ± 0.01a	0.10 ± 0.01a	0.11 ± 0.03a	0.11 ± 0.01a
	Mo (0.05)	0.21 ± 0.00a	0.20 ± 0.06a	0.12 ± 0.00a	0.12 ± 0.04a	0.17 ± 0.02a	0.18 ± 0.04a
Proteins (g/100 g)		11.64 ± 0.65a	12.52 ± 0.67a	10.43 ± 2.17a	11.15 ± 0.36a	12.56 ± 1.07a	13.19 ± 0.52a
TPC (mg GA/100 g)		26.31 ± 0.78b	67.86 ± 1.62a	2.23 ± 0.21e	8.78 ± 1.49d	10.76 ± 1.54d	16.29 ± 2.33c

Mean values in the same line sharing the same letter do not differ significantly at *p* < 0.05. TPC: Total Phenolic Compounds, LOQ: limits of quantification.

**Table 4:** Contribution of 50 g of different forms of quinoa the recommended daily intake of children, women, and men according to WHO and FAO.

Nutrient	Boiled (Bl)	Steamed (St)	Semolina (Sm)	Contribution in WHO/FAO Recommendation in (%) for:									Index
				Children Age: 4–9			Women Age: 30–51			Men Age: 30–51			
				Bl	St	Sm	Bl	St	Sm	Bl	St	Sm	
**Macro-elements**													
K (mg)	**210**	**380**	220	5.5	10.0	5.8	4.5	8.1	4.7	4.5	8.1	4.7	AI
P (mg)	170	160	60	34.0	32.0	12.0	24.3	22.9	8.6	24.3	22.9	8.6	RDA
Mg (mg)	80	80	40	61.5	61.5	30.8	25.0	25.0	12.5	19.0	19.0	9.5	RDA
Ca (mg)	40	40	40	4.0	4.0	4.0	4.0	4.0	4.0	4.0	4.0	4.0	RDA
Na (mg)	10	10	10	0.5	0.5	0.5	0.4	0.4	0.4	0.4	0.4	0.4	UL
**Micro-elements**													
Fe (mg)	1.83	1.8	1.12	18.3	18.0	11.2	10.2	10.0	6.2	22.9	22.5	14.0	RDA
Zn (mg)	0.93	0.9	0.86	18.6	18.0	17.2	11.6	11.3	10.8	8.5	8.2	7.8	RDA
Mn (mg)	0.94	0.98	0.36	62.7	65.3	24.0	52.2	54.4	20.0	40.9	42.6	15.7	AI
Cu (µg)	147.00	149.67	92.83	33.4	34.0	21.1	16.3	16.6	10.3	16.3	16.6	10.3	RDA
**Other**													
Proteins (g)	5.22	6.28	2.95	27.5	33.1	15.5	11.3	13.7	6.4	9.3	11.2	5.3	RDA

RDA = Recommended Dietary Allowance, AI = Adequate Intake and UL = Tolerable Upper Intake Level.
